# Stigma in adults with ADHD: a systematic review of types, experiences, and potential implications for quality of life

**DOI:** 10.3389/fpsyt.2026.1783271

**Published:** 2026-04-29

**Authors:** Thilaanee Krishnamoorthy, Soumitra Das, Naveen Thomas

**Affiliations:** 1 The University of Melbourne, Parkville, VIC, Australia; 2 Western Health, Melbourne, VIC, Australia; 3 Oceania University of Medicine, Apia, Samoa; 4 Deakin University, Burwood, VIC, Australia

**Keywords:** ADHD, discrimination, internalized stigma, negative labeling, perceived stigma, prejudice, public stigma, quality of life

## Abstract

**Background:**

Attention deficit hyperactivity disorder (ADHD) is a disorder characterized by hyperactive, impulsive, and/or inattentive symptoms. Adults with ADHD often report reduced quality of life (QoL) across social, educational, and occupational functioning. Part of these deficits may be attributed to stigma, which includes stereotypes, prejudices, discrimination, and negative labelling. While stigma’s effects on QoL have been extensively documented in other mental health conditions, the specific types and impacts of stigma experienced by adults with ADHD remain underexplored in recent reviews.

**Aims:**

To identify and describe the different types of stigmas experienced by adults with ADHD, while exploring how stigma may impact QoL’s key domains as defined by WHO (physical domain, psychological domain, level of independence, social relationships, environment, and spirituality/religion/personal beliefs).

**Methods:**

A literature search was conducted across APA PsycArticles, Embase, and Ovid MEDLINE(R) for ADHD AND stigma-related keywords. Eligible studies were English, peer-reviewed articles from the past decade involving adults (≥18) and describing or specifying at least one type of stigma.

**Results:**

A total of 17 papers met the inclusion criteria. Stigma types included self-stigma and/or internalized stigma, perceived stigma, public stigma, and structural stigma. QoL domains affected included the psychological domain, social relationships, environment, and level of independence. Greater ADHD symptomatology was positively correlated with more internalized stigma, which in turn was linked to functional impairment, worse self-esteem, and poorer QoL. Self-stigma manifested as self-deprecating labels and ADHD devaluation. Perceived stigma hindered treatment seeking, medication compliance, and diagnostic disclosure, although associations with QoL were insignificant. Public stigma was the most investigated and related to negative societal attitudes, notably in academic contexts. Few studies looked at structural stigma; those that did identified structural barriers to care, though none directly assessed QoL outcomes.

**Conclusion:**

Stigma remains pervasive, though direct effects on QoL domains are less widely investigated. Future studies should investigate structural stigma in more depth and explore causal relationships between stigma and QoL.

**Systematic Review Registration:**

https://doi.org/10.17605/OSF.IO/Y52HK

## Introduction

Attention deficit hyperactivity disorder (ADHD) is a childhood-onset neurodevelopmental disorder characterized by hyperactive, impulsive, and/or inattentive symptoms (1). While symptoms appear before 12, 60-86% of people diagnosed have symptoms lasting into adulthood, with literature reporting that it affects 2-6% of adults in Australia (2). Adults with ADHD report poorer self-esteem (3), inadequate healthcare access (4), and reduced quality of life across multiple domains, including social, educational, and occupational functioning (4, 5). Some deficits can be attributed to the downstream effects of the symptoms themselves, while others may stem from stigma.

Stigma includes stereotyping (negative beliefs), prejudices (negative attitudes), discrimination (negative behaviours), and labelling (6). In ADHD research, stigma refers to negative assumptions and biases towards someone based on their diagnosis and ADHD-related traits (7). Several subcategories of stigma have been identified in the literature: self-stigma, internalized stigma, perceived stigma, public stigma, and structural stigma. Self-stigma refers to the stigma an individual directs at themselves, irrespective of external factors (8). Internalized stigma is based on negative beliefs an individual holds towards themselves after internalizing societal beliefs (8). Perceived stigma refers to an individual’s worry that other people are stigmatizing them, whether true or not (9). Public stigma is stigma endorsed by society. Structural stigma involves the negative treatment of individuals through institutional policies and laws (6).

The negative impact of stigma has been well-documented in the mental health sphere. One review noted that stigma, especially internalized, hinders engagement with healthcare and therapy through shame and embarrassment (10). Another review noted the negative financial implications of mental health stigma in relation to income disparities, more expensive healthcare due to favouring investment in physical health over mental health services, and being overlooked for employment (11). Other consequences include difficulties seeking housing and impaired social relationships (12). These findings suggest stigma poses a threat to well-being, societal status, andquality of life.

Quality of life (QoL), as defined by WHO, includes 6 broad domains—the physical domain, psychological domain, level of independence, social relationships, environment, and spirituality/religion/personal beliefs. Most of these domains of QoL are further subdivided into smaller concepts, except for spirituality/religion/personal beliefs. The physical domain encompasses pain and discomfort, energy and fatigue, sexual activity, sleep and rest, and sensory functions. The psychological domain includes positive feelings; thinking, learning, memory, and concentration; self-esteem; body image and appearance; and negative feelings. Level of independence incorporates mobility, activities of daily living, dependence on medicinal substances and medical aids, dependence on non-medicinal substances, communication capacity, and work capacity. Social relationships include personal relationships, practical social support, and activities as a provider/supporter. Finally, environment has the most sub-concepts under it, which are freedom, physical safety, and security; home environment; work satisfaction; financial resources; health and social care: accessibility and quality; opportunities for acquiring new information and skills; participation in and opportunities for recreational/leisure activities; physical environment (pollution/noise/traffic/climate); and transport (13). Together, these domains and sub-concepts allow for an overarching and nuanced interpretation of quality of life.

Existing literature on ADHD-related stigma and consequences primarily focuses on pediatric populations. In reviews, stigma was demonstrated to be associated with poor medication compliance, reduced help-seeking behaviour, social exclusion by peers, and exclusionary behaviours endorsed by adults (14, 15). However, reviews on stigma in adult populations have been comparatively sparse. The limited existing literature has noted that adults with ADHD are rated to have decreased social desirability by other adults, similar to the finding of social exclusion in pediatric populations (14). However, adults face unique challenges, including navigating employment, romantic relationships and family management, and independently accessing healthcare, which may introduce novel stigma experiences than in childhood.

As far as we know, no reviews in the past decade have comprehensively synthesized data on stigma in adult ADHD. Thus, this review sought to answer the following question: how is stigma experienced by adults with ADHD, and what are its types, associated factors, and potential impact on QoL?

Our primary aim was to identify and describe the different types of stigmas experienced by adults with ADHD, with a secondary, exploratory aim to examine potential impacts of stigma on QoL across key concepts like self-concept, relationships, education, occupation, and healthcare. This review hopes to highlight the complex burden of stigma and identify potential targets for education, advocacy, and healthcare.

## Methods

### Search strategy

The databases searched between January 1, 2015, and March 13, 2025, were APA PsycArticles, Embase (1947 to 2025 March 13), and Ovid MEDLINE(R) (March 13, 2025). These databases were selected for their extensive coverage of psychiatric literature on adult ADHD. All three databases were accessed through the Ovid platform and searched simultaneously using a single combined search strategy. The following is the final search strategy using Boolean operators:

1. (“ADHD” or “attention deficit hyperactivity disorder” or “attention deficit disorder” or “attention deficit” or “attention-deficit” or “ADHD inattentive” or “ADHD hyperactive-impulsive” or “ADHD combined” or “Hyperkinetic Disorder*”).ti,ab. 125485
2. (“Prejudice” or “stigma” or “stereotype*” or “inequality” or “negative attitude*” or “perceived discrimination”).ti,ab. 248774
3. 1 and 2 1104
4. Limit 3 to english language 1066
5. Limit 4 to human 1026
6. Limit 5 to humans 912
7. Remove duplicates from 6 620
8. Limit 7 to dt=20150101–20250313 [Limit not valid in Embase,APA PsycArticles; records were retained] 608
9. Limit 8 to dd=20150101–20250313 [Limit not valid in Ovid MEDLINE(R),APA PsycArticles; records were retained] 383
10. Limit 9 to up=20150101-20250313 380

Note that the date limits from lines 7 to 10 each correspond to a single database, as each database uses a different convention to filter research articles by date. These database-specific limits were applied only to ensure consistent temporal filtering and do not otherwise modify the underlying search strategy or inclusion criteria. Only peer-reviewed articles were included to ensure a standardized quality of data. Another 2 studies were found through citation searching of relevant papers. Articles were manually screened to exclude those focused on pediatric populations (<18) to avoid inadvertently excluding relevant adult studies. QoL-related domains were identified based on the WHO domains during full-text screening and data extraction to reduce the risk of overly limiting the search strategy, especially because QoL measures are often not standardized across studies.

### Inclusion criteria

The inclusion criteria were:

- Adults (≥18) with ADHD
- Focused on self-stigma, internalized stigma, perceived stigma, public stigma, and structural stigma
- English language
- Human studies
- Published in a peer-reviewed journal in the last 10 years
- Quantitative, qualitative, and/or mixed-methods research designs

### Exclusion criteria

The exclusion criteria were:

- Children (<18)
- Non-specific descriptions of stigma unable to be recategorized by this review’s definitions
- Non-English language
- Non-human studies
- Opinion pieces, editorials, case reports, case series, newsletters, meta-analyses, and/or review articles

### Screening and data extraction

All articles found through Ovid were imported into Covidence and went through each step of title and abstract screening, full-text screening, and data extraction by 2 independent reviewers. A third reviewer took responsibility for conflict resolution. A study documentation table was created on Microsoft Excel to methodically extract information (**Table 1**). In summary, information collected from studies included publication details (i.e. authors, year of publication), methods (i.e. study design), participant demographics (i.e. age, sex, population group), stigma outcomes and outcome measures, QoL domain and sub-concepts, if present, and main findings.

**Table 1:** Summary of included studies (data extraction table).

Author(s) and Publication Year	Location	Study Design	Sample demographics (age, gender, comorbidities)	Sample size (n)	Assessment tool(s) for stigma and QoL	Type of stigma(s) specified by article	Type of stigma(s) (if not specified by paper)	QoL domain and sub-concept assessed (if present)	Main findings
Quiroz et al. (2025)(16)	USA	Quantitative (between-groups experimental)	Mean age(control)=33.83+/-8.49 n(female control)=27 n(male control)=36 n(not listed)=1 Mean age(ADHD)=35.80+/-9.45 n(female ADHD)=30 n(male ADHD)=34 n(non-binary)=1 Age range=18-69 Hispanic/Latinx participants	n(total control)=64 n(total ADHD)=65	Public Stigma Instrument Questionnaire	Public stigma			Participants endorsed significantly higher levels of public stigma toward an individual depicted to have ADHD in a clinical vignette than they did towards a control vignette with no depicted issues (3.68+/-0.82 vs.1.87+/-0.77, p<0.001).
Canu et al. (2024)(17)	USA	Quantitative (between-groups experimental design)	Mean age=19.33+/-2.67 n(female)=264 n(male)=50	n(control video)=77 n(medication mentioned only video)=77 n(symptom displayed only video)=79 n(symptom displayed and medication mentioned video)=79 n(total undergraduate students)=314	Desire for Affiliation (DFA) 3-item questionnaire Interpersonal Liking Measure-6 for degree of liking (DOL) Treatment Acceptability Questionnaire (TAQ) - Medical Treatment Subscale	Social stigma	Public stigma		On average, participants had a significantly higher DFA for the woman who did not display ADHD symptoms (6.74) and the woman using Adderall with no displayed ADHD symptoms (6.73) than the woman displaying ADHD symptoms (5.12) and the woman displaying ADHD symptoms and taking Adderall (4.95, p<0.001). Researchers also found a similar significant difference in DOL, whereby participants preferred the woman not displaying ADHD symptoms (6.49) and the woman only using Adderall (6.48) over the woman displaying ADHD symptoms (5.04) and the woman displaying ADHD symptoms and taking Adderall (5.21, p<0.001). There was no significant effect of medication for DFA or DOL: a woman with no ADHD symptoms and no medication was not scored significantly different from a woman with no ADHD symptoms taking medication, and a woman with symptoms alone was not scored significantly differently from a woman with symptoms who took medication.
Godfrey et al. (2021)(18)	Germany	Quantitative (between-groups experimental design)	Mean age(control)=27.75+/-10.88 n(female control)=74 n(male control)=43 Mean age(simulation)=27.46+/-10.87 n(female simulation)=58 n(male simulation)=47 Mean age(ADHD)=34.81+/-11.10 n(female ADHD)=35 n(male ADHD)=63	n(total control)=117 n(total simulation)=105 n(total ADHD)=98	Conners’ Adult ADHD Rating Scales (CAARS) Weiss Functional Impairment Rating Scale (WFIRS)	Public perception of adult ADHD Negative misconceptions	Public stigma		Participants in the simulation group pretending to complete the questionnaires as though they had ADHD significantly overestimated symptoms than people who had ADHD at p<0.001 in the domains of hyperactivity (26.53+/-5.50 vs. 18.79+/-7.26), DSM-V criteria for hyperactivity-impulsivity (19.21+/-4.66 vs. 12.53+/-5.58), and DSM-V total score (38.50+/-8.47 vs. 30.46 +/-8.43) on the CAARS. The simulation group also significantly overestimated functional impairment at p<0.001 in the WFIRS categories of family (1.66+/-0.54 vs.1.34+/-0.65), work (1.73+/-0.55 vs.1.16+/-0.68), school (1.92+/-0.49 vs.1.28+/-0.80), social (1.62+/-0.56 vs. 1.24+/-0.65), and risk (1.42+/-0.57 vs.0.78+/-0.52) than people with ADHD. On both the CAARS (8.36+/-3.86 vs.11.85+/-4.41, p<0.001) and WFIRS (1.53+/-0.67 vs.1.82+/-0.82, p=0.004), the simulation group significantly underestimated impairments in self-concept than people with ADHD. ADHD respondents scored significantly higher than controls on all domains of both questionnaires.
Quenneville et al. (2020)(19)	Switzerland	Quantitative (cross-sectional survey study)	Mean age (ADHD)=35.90+/-12.88 n(female ADHD)=58 n(male ADHD)=77	n(total ADHD)=135	Internalized Stigma of Mental Illness (IMSI)	Internalized stigma		Psychological domain – negative feelings Social relationships – personal relationships	Respondents with greater symptomology of ADHD based on the Adult Self-Report Scale v1.1 measure of ADHD symptoms reported greater alienation (p=0.003), stereotype endorsement (p=0.004), and social withdrawal (p=0.021) on the IMSI Scale. Increased symptoms based on the Wender Utah Rating Scale self-report of ADHD symptoms were associated with higher total score (p=0.02), alienation (p<0.001), discrimination (p=0.001), and social withdrawal (p<0.001) on the IMSI Scale.
Masuch et al. (2019)(20)	Germany	Quantitative (cross-sectional survey study)	Mean age=41.8+/-11.2 n(female)=57 n(male)=47	n(total)=104	Internalized Stigma of Mental Illness (IMSI) Questionnaire on Anticipated Discrimination (QUAD) Questionnaire on Stigmatizing Attitudes toward Adults with ADHD (QPS) Rosenberg Self-Esteem Scale (RSE) Weiss Functional Impairment Rating Scale (WFIRS) Quality of Life Satisfaction and Enjoyment Questionnaire- Short Form (Q-LES-Q-SF)	Internalized stigma Perceived public stigma Anticipated discrimination		Psychological domain – self-esteem Functional impairment measured by WFIRS (covers multiple QoL domains and sub-concepts) QoL measured by Q-LES-Q-SF	Only 23.3% of participants reported high internalized stigma, and overall mean scores on IMSI were below threshold for high levels of internalized stigma. Many participants (88.5%) noted high anticipated discrimination in minimum one area, with the highest prevalence from employers (>70%), colleagues (>60%), housing (>50%), benefits (>50%), education (>50%), and neighbourhood (>50%). Many participants perceived public stigma (69.3%). Perceived public stigma was notable in the areas such as “Ability to Take Responsibility” (74%), “Norm-Violating and Externalizing Behaviour” (66%), “Reliability and Social Functioning” (65.4%), and “Malingering and Misuse of Medications” (56.7%). Key examples of perceived public stigmas included “ADHD is a childhood disorder not seen in adults” (88.5%),”Adults with ADHD act without thinking” (87.3%), “ADHD is caused by bad parenthood” (82.4%), “ADHD is invented by drug companies” (76.0%), “ADHD is caused by extensive exposure to video games or TV shows” (73.3%), and “Adults with ADHD simulate their symptoms” (72.8%). Internalized stigma was moderately positively correlated with ADHD symptoms (r=0.46, p<0.001) and functional impairment (r=0.61, p<0.001) and moderately negatively correlated with self-esteem (r=-0.64, p<0.001) and quality of life (r=-0.56, p<0.001). Anticipated discrimination was weakly positively correlated with functional impairment (r=0.26, p<0.05) and weakly negatively correlated with quality of life (r=-0.21, p<0.05). Results demonstrated no significant relationships between perceived public stigma and self-esteem, functional impairment, or quality of life.
Speerforck et al. (2019)(21)	Germany	Quantitative (cross-sectional experimental design)	German-speaking adults Slightly more women	n(total ADHD adult)=505	Emotional Reactions to the Mentally Ill Scale (ERMIS) The desire for social distance scale	Stigmatizing attitudes	Public stigma		After watching a vignette of an individual with adult ADHD, 24% of participants agreed to feeling annoyed by, 17% agreed they felt uncomfortable with, and 13% agreed they reacted angrily to the individual in the vignette. Overall, however, many participants disagreed with these negative emotional reactions to the individual with adult ADHD. In terms of social distance, over 40% of participants rejected having an individual with depicted ADHD care for their children compared to ~30% acceptance. In other domains, like renting a room to someone with ADHD (~50% acceptance vs ~20% rejection) and working together with someone with ADHD (74% acceptance vs ~10% rejection) participants had more acceptance over rejection. Recommending someone with ADHD for a job had greater acceptance than rejection, but only by ~10% (34% acceptance vs ~25% rejection).
Foy (2018)(22)	USA	Quantitative (experimental)	Mean age=25.31 +/-7.31 (ANCOVA limited to ages 18-24) % female=61% % male=39%	n(ADHD stereotype threat)=27 n(ADHD no stereotype threat)=26 n(no ADHD stereotype threat)=30 n (no ADHD no stereotype threat)=31 n(total)=114	Graduate Record Exam (GRE92-1) - subset of verbal and quantitative questions --> analyzed scores after exposure to stereotype threat	Stereotype threat	Public stigma	Psychological domain – thinking, learning, memory, and concentration	Overall, participants with ADHD had lower average scores on both verbal questions (6.72 vs 8.21, p<0.001) and quantitative questions (4.85 vs 8.74, p<0.001) than people without ADHD. In quantitative questions, people with ADHD exposed to the stereotype that people with ADHD did poorer on the assessment performed significantly worse (4.19) than people with ADHD not exposed to this stereotype threat (5.54, <0.05). However, in verbal questions, the researcher found no significant difference between people with ADHD exposed to the stereotype threat (6.81) and people with ADHD not exposed to the stereotype threat (6.62, p>0.05).
Thompson et al. (2016)(23)	USA	Quantitative (experimental)	Phase 1 Mean age=18.66 Age range=18-25 % female=70.4% Phase 2 Mean age=18.94 Age range=18-22 % female=64.6% Non-ADHD participants	n(Phase 1 total)=135 n(Phase 2 total)=48	Anticipated Behaviour Form (ABF) Social Distance Scale (SDS)	Not explicitly stated, but measured college students’ level of stigma towards ADHD label and behaviours	Public stigma		Behaviours typical of ADHD as represented on a falsely completed information form were significantly associated with lower mean scores on the ABF (p<0.001), suggesting increased stigma. Researchers found no significant association between the label of ADHD and ABF scores, behaviours of ADHD and SDS scores, and the label of ADHD and SDS scores. There were no significant interaction effects of behaviours of ADHD and label of ADHD on both the ABF and SDS scores. No significant difference was found on the mean ABF and SDS scores between depression and ADHD.
Aksoy et al. (2017)(24)	Turkey	Quantitative (cross-sectional survey study)	Psychiatrists in Turkey Mostly male	n(psychiatrists)=85	Questionnaire evaluating the beliefs of psychiatrists on adult ADHD	Not explicitly stated, but measured psychiatrist’s attitudes towards adult ADHD	Public stigma		A greater proportion of psychiatrists tended to endorse more positive than negative attitudes towards adult ADHD. However, there were a subset who endorsed negative, stigmatizing attitudes. One statement endorsed by over 1/3 of psychiatrists was “I fear of the abuse potential of stimulant prescriptions and am uneasy in prescribing them” (1.2% strongly agreed, 34.1% agreed). A subset of psychiatrists believed that “Stimulant drugs lead to dependence” (4.7% strongly agreed, 22.4% agreed). While many psychiatrists agreed that ADHD is real (91.8%), 4.8% of psychiatrists endorsed the statement “There is no such disorder as ADHD.” Furthermore, 12.9% of psychiatrists agreed that “ADHD is a disorder of childhood and its signs and symptoms usually remit in adulthood.” A small percentage (3.5%) agreed to the statement that “Signs of ADHD do not affect life and as such are unimportant,” though the majority either disagreed (28.2%) or strongly disagreed (68.2%).
Babinksi et al. (2025)(25)	USA	Mixed-methods	Mean age=39.43+/-6.37 Age range=31-52 %female=100%	n(total)=14	Focus group recordings - analyzed via Interpretative Phenomenological Analysis (IPA) framework	Not explicitly stated, but themes included difficulty accessing care, burden of care, and stigma and isolation	Public stigma Structural stigma	Environment – health and social care: accessibility and quality	In terms of experiences with doctors, a few women noted how doctors invalidated their diagnosis of ADHD and attributed their symptoms to other mental health conditions including PTSD, BPAD, anxiety, and depression. One woman using stimulant medications experienced a doctor stating “Oh, no, you have substance abuse problems. You have to go to rehab.” Another woman perceived doctors to have dismissive attitudes in minimizing the condition as commonplace and limiting their time with her during consults. Regarding the effects of stigma, one woman reported reluctance to test her child due to fear of medication stigma. Related to structural barriers, a few women reported difficulty accessing doctors who were willing to treat their ADHD after their initial diagnosis and treatment by one doctor. Several women were limited by their insurance providers in terms of accessing the health professionals they needed like a psychologist or other health practitioners adequately equipped to manage ADHD. The diagnostic process itself was noted by one woman to be exceptionally long.
Cao et al. (2024)(26)	Australia	Mixed-methods (cross-sectional)	Psychiatrists in South Australia	n(psychiatrists seeing all adults)=31 n(psychiatrists seeing mostly adults)=27 n(psychiatrists seeing equal numbers of children and adults)=6 n(psychiatrists seeing mostly children)=7 n(psychiatrists seeing all children)=2 n(total psychiatrists)=73	Online survey - collected data included attitudes and opinions about ADHD and its treatment, referral patterns and approaches to treatment, and barriers to seeing adult patients with ADHD	Not explicitly stated, but measured psychiatrist’s attitudes towards adult ADHD	Public stigma Structural stigma		58.90% (43/73) of psychiatrists responded “no” to the question “Would you accept new patients for assessment and diagnosis of ADHD in an adult?” and 49.32% (36/73) of psychiatrists responded no to the question “Would you accept referrals for continuing management of an adult who has an existing diagnosis of ADHD?”. Among the recorded barriers to seeing adult patients with ADHD, those related to stigma include “Drug-seeking behaviour of patients” (31.5%), “Potential for abuse of stimulant medications prescribed” (27.4%), “Model of care/service guidelines prevent it” (17.8%), “Concern about potential damage to my profession and clinic reputation if I did” (15.1%), and “I disagree it’s a valid diagnosis” (6.9%). In the qualitative analysis, similar themes emerged as barriers to treatment such as “lack of service availability”, including psychiatric services, GPs, and psychologists, “ADHD as a diagnosis”, which covered underdiagnosis/overdiagnosis of ADHD, and “stimulants as a treatment for ADHD”, which discussed underuse/overuse of stimulants and skepticism towards/advocacy for their use as treatment based on evidence.
Kreider et al. (2024)(27)	USA	Qualitative (descriptive design)	Mean age=21.2+/-3.5 Age range=18-33 n(female)=24 n(male)=26 n(gender not reported)=2 n(learning disability(LD))=22 n(ADHD)=18 n(LD+ADHD)=12	n(total)=52	Group meeting transcripts - analyzed for stigma via Person-Environment-Occupation-Performance Model (PEOP)	Perceived misconceptions Stigmatizing actions	Public stigma Self-stigma	Psychological domain – thinking, learning, memory and concentration; self-esteem; negative feelings Environment – opportunities for acquiring new information and skills	Family, peers, and teachers perceived participants as less intelligent than those without LD (learning disability)/ADHD. Similarly, participants themselves reported feeling inferior to their peers without LD/ADHD and juggled self-doubt about being mistakenly admitted to their reputable universities. People assumed that those with LD/ADHD could be cured by trying harder or medications, which exacerbated negative thoughts and poor self-image experienced by participants. Participants dealt with perceptions that their diagnosis was fake because of the lack of physical proof of its existence, and that their needs for educational accommodations on assessments were seen as a “cop out, an excuse, and an unfair advantage that enabled them to manipulate the system”. When they performed well academically, the dismissal of their diagnosis and their need for further support intensified. Requests for accommodations to instructors could be met with resistance and a negative change in how their instructors treated them. Medication stigma came up in discussions as the shame associated with taking medications. In relation to stigmatizing actions, participants reported discrimination from teachers who negatively generalized their past experiences with and preconceived ideas about someone with LD/ADHD to the participant. This subsequently led to participants becoming more uneasy and less engaged in their classes. Unfavourable media representations of LD/ADHD encourage stereotypes and misconceptions of their condition. Participants commonly noted downplaying of their condition by family, peers, and teachers, such as using their diagnosis as an adjective to describe inability to focus.
Patrickson et al. (2024)(28)	Australia	Qualitative (semi-structured interviews)	Age=18+ n(female)=6 n(male)=8 n(non-binary)=1	n(consumers)=9 n(health professionals)=6 n(total)=15	Semi-structured interview	Ignorance and prejudice Not explicitly stated, but health practitioner beliefs	Public stigma	Environment – health and social care: accessibility and quality	Consumers with ADHD noted ignorance and prejudice in the context of their GP (general practitioner) refusing to take them seriously when they tried to get their ADHD diagnosed. One individual said “Some GPs dismissed me, saying, ‘You wouldn’t have ADHD, otherwise you wouldn’t be able to manage to work and all that’”, and another individual reported “because of my past with substance abuse and alcohol, my GP kinda brushed it [diagnosis of ADHD] off at first”. One health practitioner claimed they had a “lingering worry about the potential for medication abuse”.
Aoki et al. (2020)(29)	Japan	Qualitative (semi-structured interviews)	Mean age=36.5 Age range=23-55 n(female)=6 n(male)=6	n(total)=12	Semi-structured interview	Self-stigma Labelling		Psychological domain – self-esteem, negative feelings	Participants did not like that the condition was referred to as a “developmental disorder”, as they held negative views of what they thought a developmental disorder looked like. Key phrases expressing their views include “lack of development and looking stupid” and “oddballs”. The interviewees had both been labelled and labelled themselves as “inferior” to their peers, and the words “sloppy” and “lazy” came up as descriptors.
Khan et al. (2021)(30)	Australia	Qualitative (focus group discussions)	Median age=34+/-11.5 n(female)=9 n(male)=20	n(total)=20	Focus group recordings - analyzed via framework for thematic analysis	Negative beliefs Medication stigma	Perceived stigma	Level of independence – dependence on medicinal substances and medical aids	Participants held negative beliefs towards stimulant medications for ADHD due to their distrust of pharmaceutical companies and worries about side effects. As adults, fear of workplace stigma from colleagues regarding medication use impacted their medication compliance. One participant noted they’d “be seen as a bad person to other people so it’s like the stigma attached to the medication”.
Watters et al. (2018)(31)	Ireland	Qualitative (semi-structured interviews)	Mean age=37.64+/-11.83 Age range=20-54 n(female)=2 n(male)=9	n(total)=11	Semi-structured interview	Perceived stigma towards mental health from ADHD participant and family	Perceived stigma Public stigma	Psychological domain – negative feelings Environment – health and social care: accessibility and quality	One participant noted reluctance to get medical treatment from a hospital psychiatrist for fear of being seen as not “ok”. They also noted their family’s difficulty in accepting a mental health condition over a physical health condition. Many participants reported being misinformed about ADHD in thinking that it was only a childhood condition, having impressions it was “kids just throwing tantrums” or “unruly kids”. A participant mentioned the idea of people debating whether ADHD was true or an invented diagnosis as “an excuse for boldness”. Another concept that came up was the idea of media portrayals of ADHD reserved for stereotypically bad characters, which further enabled ADHD to be viewed negatively.
Holthe (2017)(32)	USA	Qualitative (semi-structured interviews)	Age range=32-50 % female=100%	n(total)=5	Semi-structured interview	Not explicitly stated, but qualitatively noted types of stigma experienced	Perceived stigma Public stigma Self-stigma	Psychological domain – self-esteem, negative feelings	The women reported noticing public stigma towards ADHD in media and articles and stigma towards themselves from family and friends. They reasoned that this stemmed from misunderstandings about ADHD, minimization of the disorder, and negative opinions and media regarding whether the diagnosis and treatment are valid. They further attribute stigma to the fact that as a mental health condition, the disability is “invisible”. Some women maintained negative self-image into adulthood due to their negative perceptions of themselves as lazy and incapable. The lack of quantitative investigations for ADHD also led to self-doubt about its validity in these individuals. Fear of stigmatization led the women to keep their diagnosis private, including from colleagues at their workplace, and only entrust their diagnosis to close family and friends. The women noted gender differences, where they felt men with traits like impulsivity, disorganization, and high energy were seen more favourably than women.

### Risk of bias

Risk of bias was assessed by two independent reviewers using either the JBI critical appraisal tools (quasi-experimental studies, analytical cross-sectional studies, or qualitative studies) or the Mixed Methods Appraisal Tool based on the study design. A third reviewer was responsible for resolving conflicts. Generally, most of the included articles had a low to moderate risk of bias. Aksoy and Tufan (24) was the only study rated to have a high risk of bias.

### Registration

This review protocol was retrospectively registered with the Open Science Framework to enhance transparency (https://doi.org/10.17605/OSF.IO/Y52HK).

## Results

**Figure 1:**
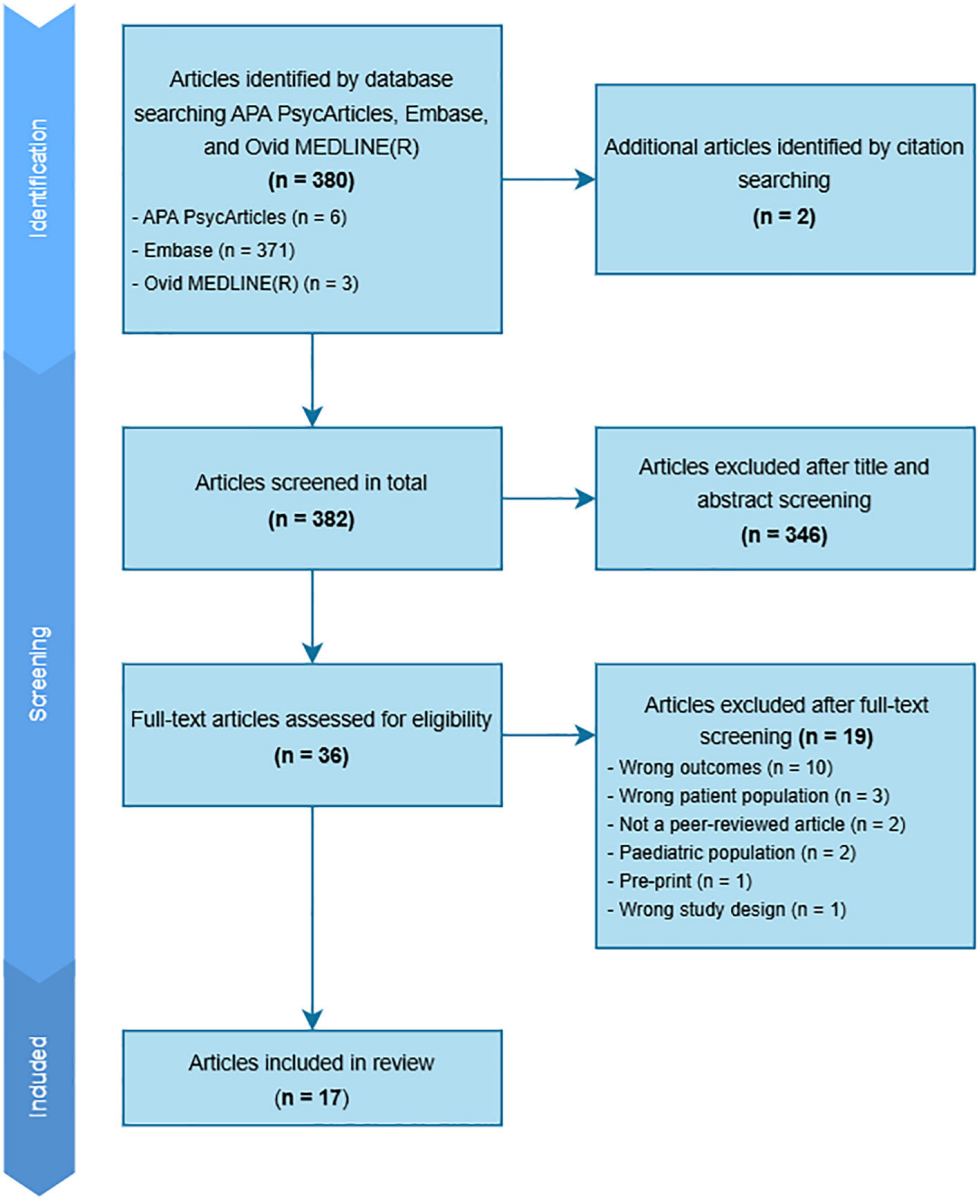
PRISMA Flow Diagram.

The complete article review process is detailed in a PRISMA Flow Diagram (**Figure 1**). To summarize, the search strategy yielded 380 articles that were imported into Covidence and 2 articles identified through citation searching. After title and abstract screening and full-text screening, 17 articles were included in this review. Full details on all included studies are outlined in **Table 1**. Of the 17 articles included in the final review, 9 were quantitative, 2 were mixed-methods, and 6 were qualitative. Regarding where each study was conducted, 7 were from the USA, 3 were from Australia, 3 were from Germany, 1 was from Ireland, 1 was from Switzerland, 1 was from Turkey, and 1 was from Japan. Participants across all studies ranged from ages 18 to 69, and the number of participants included in each study ranged from 5 to 505.

Articles were subcategorized according to the 5 types of stigma investigated as defined earlier, which are self-stigma, internalized stigma, perceived stigma, public stigma, and structural stigma. QoL sub-concepts affected were discussed under the relevant stigma subcategory if present.

## Discussion

### Self-stigma

Of all the studies, 3 aligned with the definition of self-stigma. Self-stigma came up in a few studies that identified participants endorsing self-deprecating labels like “lazy,” “inferior” to one’s peers, and “incapable” or “stupid” (27, 29, 32). While these were not explicitly tied to worse QoL outcomes, they are markers of poor self-esteem—a variable that has been tied to poor QoL in people with other mental health conditions (33) and is one of the sub-concepts under WHO’s psychological domain Considering that current ADHD management primarily targets symptom reduction through medication, these findings raise questions about whether individualized, self-esteem-focused interventions could play a larger role in ADHD management and functional outcomes.

In terms of issues related to ADHD labelling, individuals mentioned that they wanted to distance themselves from the words “neurodevelopmental disorder” due to their negative views of this label (29). Some also expressed self-doubt about the validity of their diagnosis given the lack of quantitative investigations (32), which may impact help-seeking behaviour if they do not believe in the legitimacy of their condition. This self-stigmatizing line of thinking might stem from a limited understanding of what ADHD means as a medical diagnosis, as well as comparisons to the work-up for physical ailments. This issue may be addressed through strategies such as increased education. For instance, neurodevelopmental disorders may be perceived as inflexible deficits rather than a spectrum (34, 35). Offering individuals a clear definition of the fluid nature of neurodevelopmental disorders using neutral terminology may offer greater insight into ADHD and reduce identity conflicts. Additionally, it may help to reaffirm that while mental health disorders may be confirmed differently than physical ones, the vast body of literature on the credibility of ADHD can help reinforce that their diagnosis is indeed valid.

### Internalized stigma

In total, 2 of the 17 studies fit the definition of internalized stigma. One pattern identified by the articles was that greater ADHD symptomatology is positively correlated with greater levels of internalized stigma (19, 20). Looking closer at the areas most significantly impacted by internalized stigma using the Adult Self-Report Scale v1.1 (ASRSv1.1) and the Wender Utah Rating Scale (WURS), internalized stigma was consistently associated with social withdrawal and alienation across both scales (19). As ADHD seems to be associated with social impairments in adults (4, 5), it would be helpful to explore the extent to which internalized stigma mediates this. Stereotype endorsement was only positively associated with the ASRSv1.1 and discrimination was only positively associated with the WURS (19). This inconsistency suggests that each scale may be assessing different facets of ADHD symptomatology that are missed by the other. It may complicate our understanding of how internalized stigma presents itself and could necessitate the creation of a standardized measure of ADHD symptom severity.

Internalized stigma was tied to functional impairment, worse self-esteem and self-image, and poorer QoL (19, 32). However, only 23.3% of participants were found to have high levels of internalized stigma, while most were subthreshold (19). Since this study did not look at their duration since diagnosis, the low reports of internalized stigma might be because there was not enough time to internalize societal attitudes. Alternatively, it could be because ADHD is being increasingly diagnosed in adults (36), leading to greater recognition and generating more available information on it, such as through social media. More public knowledge could lead to lower levels of stigma that can be internalized, but it would be important to monitor for the spread of misinformation.

### Perceived stigma

Only 4 articles discussed perceived stigma. Some studies found that perceived stigma restricts treatment-seeking behaviours, medication compliance, and diagnostic disclosure. These perceived stigmas were believed to come from colleagues who would look at the individuals as “bad people” for taking medication or anticipated public opinions of an individual being “not okay” for seeking psychiatric care (30, 31). Reluctance to seek treatment because of perceived stigma can cause care delays, which may result in untreated deficits exerting a prolonged negative impact on overall functioning. As these stigmas also stem from different areas of life, such as at work or in healthcare settings, it may underscore the need to look at anti-stigma interventions holistically to address the various environments that shape their existence.

Interestingly, one perceived public stigma that “ADHD is invented by drug companies” (20) was comparable to the mistrust that some individuals with ADHD held toward pharmaceutical companies (30). These views may indicate that stigma extends past ADHD itself and encompasses part of the broader system designed to manage it. This mistrust could pose a greater risk if it generalizes to mistrust of clinicians and mental health services. These results suggest the importance of confronting stigma early in the diagnostic and treatment process. This may prevent disengagement with services designed to help and the ensuing deterioration in mental health that comes with disengagement, thus potentially halting the worsening of long-term QoL outcomes.

Another element of perceived stigma mentioned was the gendered difference noted in one study, where women felt that traits like impulsivity, disorganization, and high energy were seen more favourably in men than in women (32). There may be some truth to this idea given that the diagnostic ratio for ADHD often favours men over women across many studies (37). Women may also have less symptom severity for hyperactive-impulsive symptoms than the more explicit symptoms displayed by men, and can often present with the more often missed inattentive symptoms (38). The perception may also be rooted in traditional gender roles endorsed by society, where women have long been expected to behave calmly and reservedly compared to men, and it is seen as a moral failing if they cannot meet these expectations. This view may exacerbate stigma, thus possibly leading to increased feelings of shame and consequently downplaying or denying symptoms. If this results in underdiagnosis, women may miss out on timely intervention and education on stigma reduction and create an ongoing relationship of perceived stigma to poor treatment seeking and vice-versa. Therefore, it may be helpful to incorporate gender-sensitive nuance regarding symptoms into awareness campaigns targeting key figures like clinicians and educators. These measures may help key figures better identify and make the appropriate referrals for women with ADHD.

In terms of studies specifically examining the relationship between perceived stigma and QoL outcomes, one study based on self-reports found no significant associations between perceived stigma and outcomes like self-esteem or quality of life (20). This was a surprising finding, as another paper investigating this relationship in other psychiatric conditions has found a meaningful association between the two (39). These findings do not necessarily mean that perceived stigma has minimal impact—it might suggest that perceived stigma works in long-term ways that cannot be measured by the cross-sectional nature of the study. It might even mean that people developed coping mechanisms to mediate stigma’s negative effects. For example, a few studies have identified the role resilience might play in stigma reduction (40–42). It would be interesting in the future to review the evidence base of what specific variables may nullify the effects of stigma in adult ADHD.

### Public stigma

Public stigma was the most widely represented in the literature, with 13/17 studies mentioning it. Across the studies, this stigma was found to be perpetrated by family, friends, peers, colleagues, employers, health professionals, and the media.

Several studies using clinical vignettes found that adults displaying ADHD symptoms were consistently viewed less favourably than adults not showing symptoms. This was found in the context of statistically significant public stigma, lower social desirability, worse anticipated behaviour, and lower desire for affiliation (16, 17, 23). Thompson and Lefler (23) demonstrated that this was not related to the label of ADHD, and Canu et al. (17) showed that this was not significantly affected by taking stimulant medications. These findings could inadvertently reinforce the pressure to mask symptoms and contribute to internalized stigma as people with ADHD are conditioned to believe their behaviours will lead to social rejection. Having said that, if stigma is triggered more by observable behaviours than explicit labels or medication use, this could have positive implications for diagnostic disclosure and treatment-seeking behaviour. People may be able to confidently disclose their ADHD status without significant social detriment or could openly medicate themselves in environments such as work, therefore removing these factors as obstacles.

Though most vignette-based studies demonstrated noteworthy stigma, Speerforck et al. (21) found that while there were individuals who endorsed negative emotional reactions towards someone displaying ADHD symptoms, most did not. This paper also noted participants reported increased social acceptance by endorsing positive statements such as a willingness to work with, rent a room to, and recommend a job to someone with ADHD. Nonetheless, rejection was more common towards the idea of someone with ADHD being allowed to care for their child. Taken together, these results could highlight that public stigma may be context-dependent and more strictly applied in sensitive environments. It might be useful for future studies to explore this nuance in greater detail to determine if public stigma lies on a continuum.

Various overlapping stigmas emerged when exploring health practitioner perspectives, particularly surrounding fears of stimulant abuse and drug-seeking behaviour, minimization of ADHD symptoms and the resulting impairments they cause, and questioning the overall validity of the diagnosis (24–26, 28). These views are concerning given that clinicians are the first point of entry for patients into ADHD diagnosis and treatment. Holding baseless assumptions towards patients and letting them seep into clinical interactions may permanently damage the therapeutic relationship and shape how patients view the health system. This may subsequently lead to mistrust in health practitioners and avoidance of care, furthering the burden of shame.

Both experimental and qualitative studies demonstrated the influence of public stigma in academic contexts. Foy (22) found that individuals with ADHD exposed to the stereotype “people with ADHD performed worse on this assessment” performed worse on the quantitative section of the assessment than those not exposed. Intriguingly, these same differences were not seen in the verbal section. One explanation may be that people commonly have baseline anxiety around math and quantitative reasoning, and the explicitly stated stereotype could have validated this doubt, further impeding performance through a self-fulfilling prophecy. However, without knowing the participants’ educational strengths, this explanation remains speculative. Qualitative findings by Kreider et al. (27) noted public stigma affected perceptions of intelligence and caused individuals to be questioned regarding their need for examination accommodations. Performing well further reinforced public doubts about the legitimacy of their diagnosis. Negative misconceptions endorsed by professors based on past experiences of students led to altered behaviour towards these students, consequently resulting in participants becoming more uneasy and less engaged in their classes. It should be acknowledged that this study combined results from individuals with ADHD and/or learning disabilities, so the results may not have accurately captured the academic deficits central to ADHD. Nevertheless, over time, the continued invalidation of ADHD and negative stereotypes may contribute to learned helplessness, where adults with ADHD disengage academically and consistently underachieve, not because they are incapable but because of internalized doubts.

### Structural stigma

Only 2 out of 17 studies hinted at themes of structural stigma, making this the least represented stigma in the literature. However, both studies raised valid concerns about structural barriers warranting further research.

Both studies addressed the aspect of difficulty accessing care due to limited availability of GPs, psychiatrists, and psychologists; clinicians’ unwillingness to accept patients with ADHD; insurance barriers in providing coverage for the relevant health professionals; and diagnostic delays (25, 26). Cao et al. (26) noted that nearly half the psychiatrists surveyed responded no to the question “Would you accept referrals for continuing management of an adult who has an existing diagnosis of ADHD?”, aligning with Babinski and Libsack (25) reporting that women with ADHD were unable to find a provider willing to treat them after moving away from the doctor who initially diagnosed them. The women in one study also reported that the therapists themselves did not have adequate knowledge of how to manage patients with ADHD (25). These findings may point to individual discrimination but may also indicate a defect in professional education programs. Addressing these structural issues may necessitate change at institutional levels. For example, regarding the limited availability of appropriate clinicians and educational gaps, key stakeholders could provide monetary incentives whereby clinicians specializing in ADHD care are offered higher salaries. They could also design and implement educational training programs that include more theory and practical experience managing adults with ADHD.

The lengthy diagnostic timelines described by some women may be connected to the idea of limited service providers or can be its own issue based on stigma towards adult ADHD. The challenge related to diagnosing an individual with a neurodevelopmental disorder in adulthood may stem from conservative diagnostic criteria and reduced allocation of resources for diagnosis. Similarly, insurance restrictions limiting access to qualified health professionals may reflect resource allocation to cost-efficient services over adequate ADHD support. This may introduce financial barriers, whereby lower-income individuals have fewer opportunities to receive comprehensive care than higher-income individuals.

Cao et al. (26) mentioned one of the structural-leaning barriers to care that emerged was the “model of care/service guidelines prevent it” as part of the reason psychiatrists would be unwilling to take new adult ADHD referrals. Though not explicitly elaborated upon in the study, the models and/or service guidelines may involve an undervaluing of adult ADHD in mental health services in favour of more acute psychiatric presentations, or barriers related to stringent prescribing policies. However, this lack of transparency may itself suggest that the guidelines are poorly written, making it difficult to pinpoint their discriminatory nature.

It is important to acknowledge that both studies focused on specific populations—Babinski and Libsack (25) focused on women and Cao et al. (26) only included psychiatrists, therefore meaning that the studies were not representative of the general adult ADHD population. The scarcity of literature on structural stigma and its direct causal relationship with poor QoL outcomes highlights a serious gap in the literature. Structural stigma can be harder to measure because it is embedded in institutional policies and practices, and not just explicit individual attitudes. It may be easier to look at downstream consequences like unequal access to services or lack of accommodations, but these aren’t always captured in standard stigma scales or self-report studies. We need more research to uncover how institutional policies contribute to stigma, such as future studies reviewing unintentionally discriminatory guidelines and policies.

### Limitations

Several limitations were identified with this review. Firstly, this review only included studies from three databases (APA PsycArticles, Embase and Ovid MEDLINE(R)). While these databases comprehensively cover psychiatric literature on adult ADHD, limiting the search to three databases could have excluded relevant articles indexed in other databases. Another limitation relates to the search strategy, which relied primarily on free-text title and abstract terms rather than database-specific controlled vocabulary (e.g. MeSH in MEDLINE or Emtree in Embase). The absence of controlled vocabulary may have reduced the sensitivity of the search and increased the possibility that some relevant studies were not retrieved; however, broad keyword combinations were used to minimize this risk. Next, stigma terminology was used inconsistently across studies, limiting comparability and potentially affecting synthesis. Additionally, most studies did not have a standardized measure of QoL, and most only assessed the stigma experienced by a person with ADHD without assessing its downstream effects on their QoL, requiring indirect inferences regarding the relationship between stigma and QoL. These factors combined introduced bias as the studies were recategorized in terms of stigma and QoL using this review’s definitions. Next, most studies were correlational or cross-sectional, meaning no causal statements could be made on whether stigma caused the reported negative outcomes. Finally, there was a strong Western bias, with minimal cultural diversity represented, limiting the overall generalizability of the findings to other communities.

## Conclusion

In summary, this review aimed to examine the types and experiences of stigma in adults with ADHD and explore any potential impacts of stigma on various QoL domains. The first aim was mainly met through qualitative and quantitative research, offering a detailed exploration of self stigma, internalized stigma, perceived stigma, and public stigma, with less exploration of structural stigma. The secondary exploratory aim regarding QoL was not met as thoroughly, primarily due to the limited number of studies utilizing direct, standardized measurements of stigma and QoL outcomes. Overall, the findings suggest that stigma remains burdensome and affects overall functioning and well-being across many contexts. Future research could explore structural stigma and domains of QoL in more depth; include more randomized, controlled-designed studies; examine stigma differences across ADHD subtypes; and expand into non-Western populations to ensure findings are globally relevant.

## Data Availability

The original contributions presented in the study are included in the article/supplementary material. Further inquiries can be directed to the corresponding author.
